# A243 BONE MINERAL DENSITY AND DIETARY BONE NUTRIENTS DIFFER IN PATIENTS WITH CROHN'S DISEASE STRICTURES

**DOI:** 10.1093/jcag/gwad061.243

**Published:** 2024-02-14

**Authors:** A Macci, J Basit, R Klassen, R E Rosentreter, K Bindra, B Lethebe, S Boyd, E Billington, M Raman, L Burt, C Lu

**Affiliations:** Medicine, University of Calgary Cumming School of Medicine, Calgary, AB, Canada; Medicine, University of Calgary Cumming School of Medicine, Calgary, AB, Canada; Medicine, University of Calgary Cumming School of Medicine, Calgary, AB, Canada; Medicine, University of Calgary Cumming School of Medicine, Calgary, AB, Canada; Medicine, University of Calgary Cumming School of Medicine, Calgary, AB, Canada; University of Calgary, Calgary, AB, Canada; University of Calgary, Calgary, AB, Canada; Alberta Health Services, Calgary, AB, Canada; Medicine, University of Calgary Cumming School of Medicine, Calgary, AB, Canada; University of Calgary, Calgary, AB, Canada; Medicine, University of Calgary, Calgary, AB, Canada

## Abstract

**Background:**

Crohn’s disease (CD) phenotypes include inflammatory (non-stricture) and stricture behaviours. Strictures are bowel narrowing’s that may lead to obstructions and subsequent dietary restrictions to prevent blockages. Bone mineral density (BMD) in CD is affected by many factors including corticosteroid use, CD itself, but also dietary changes. Clinical gold standard for measuring BMD is by dual x-ray absorptiometry (DXA) scans. High-resolution peripheral quantitative computed tomography (HR-pQCT), a more precise imaging modality assesses volumetric BMD, bone geometry and bone microarchitecture. We aim to compare dietary intake, and bone quality in both stricture and non-stricture CD.

**Aims:**

To identify nutritional patterns, bone quality differences and deficiencies between two subtypes of CD; including energy intake, dietary components and/or micronutrients

**Methods:**

Individuals (≥55 years old) with ileal CD strictures (fixed, angulated bowel or thickened bowel with pre-stenotic dilation on diagnostic imaging), or inflammatory CD were prospectively recruited. Patients with short gut, celiac disease, dysphagia history were excluded. All patients completed DXA hip and spine scans and HR-pQCT radius and tibia scans. Dietary assessment questionnaires (Automated Self-Administered 24-hour Dietary Assessment Tool (ASA24), and Diet History Questionnaire III for food frequency over the past year were completed.

**Results:**

56 (27 inflammatory (48% female, mean age 64.2, BMI 28.7, 16 years CD duration) and 29 strictures (45% female, mean age 65.9, BMI 27.3, 25 years CD duration, 15 (51.7%) prior bowel resection) were included. 31/56 (55.4%) patients were on biologics and 11 (19.5%) had past corticosteroid exposure. DXA-based BMD for stricture versus non-stricture CD patients was not significantly different at the hip or spine. However, HR-pQCT-based radial total BMD (p=0.03), cortical thickness (p=0.01) and cortical area (p=0.04) were lower in stricture than non-stricture patients.11 (7 stricture, 4 inflammatory) patients with past steroids consumed more dairy (p=0.01), vitamin D (p = 0.04), and calcium (p=0.03) than other CD patients. Stricture patients had significantly less consumption of vitamin K (p=0.001), vegetables (p=0.02), calcium (p=0.03), and dairy (p=0.03).

**Conclusions:**

Patients with CD strictures have lower BMD and cortical bone quality at the radius than those without strictures. Patients with CD strictures also consume significantly less vitamin K, calcium, and dairy, all vital dietary components to prevent OP, compared to non-stricture patients. Overall, the skeletal fracture risk is likely much greater in CD patients with strictures than without, due to greater inherent bone fragility, and less consumption of key nutrition building blocks of bones.

**Funding Agencies:**

Koopmans Memorial Research Fund

